# Supplementary material for: Femtosecond proton transfer in urea solutions probed by X-ray spectroscopy
Source: Nature. 2023 Jun 28;619(7971):749–54. doi: 10.1038/s41586-023-06182-6 (PMC10371863; doi:10.1038/s41586-023-06182-6)
Supplement: Supplementary file 1 — Additional results sections 1–1.9, including Supplementary Figs 1–11. [file 41586_2023_6182_MOESM1_ESM.pdf]

nature portfolio
https://doi.org/10.1038/s41586-023-06182-6

Supplementary information

Femtosecond proton transfer in urea solutions probed by X-ray spectroscopy

In the format provided by the authors and unedited

Supplementary Information for Femtosecond Proton Transfer in Urea Solutions Probed by X-ray Spectroscopy

Zhong Yin[1*†], Yi–Ping Chang[2†], Tadas Balčiūnas[1,2†], Yashoj Shakya[3,4†], Aleksa Djorović[2], Geoffrey Gaulier[2], Giuseppe Fazio[1], Robin Santra[3,4,5], Ludger Inhester[3,5*], Jean–Pierre Wolf[2*] and Hans Jakob Wörner[1*]

[1]Laboratory for Physical Chemistry, ETH Zürich, 8093 Zürich, Switzerland.
[2]GAP–Biophotonics, Université de Genève, 1205 Geneva, Switzerland.
[3]Center for Free-Electron Laser Science CFEL, Deutsches Elektronen-Synchrotron DESY, 22607 Hamburg, Germany.
[4]Department of Physics, Universität Hamburg, 22607 Hamburg, Germany.
[5]Hamburg Centre for Ultrafast Imaging, Universität Hamburg, 22607 Hamburg, Germany.

*Corresponding author(s). E-mail(s): yinz@tohoku.ac.jp; ludger.inhester@desy.de; jean-pierre.wolf@unige.ch; hwoerner@ethz.ch;
†These authors contributed equally to this work.

1 Additional Results

1.1 Urea Aggregation

Figure S1 illustrates the aggregation pattern of urea molecules in high-concentration aqueous solutions. From snapshots of the MD simulation, we calculated the concentration of different urea aggregates linked via hydrogen bonds. The concentration of

monomers, dimers, and larger oligomers is shown in Fig. S1a for the two considered concentrations, namely 10 M and 5 M. As can be seen, for both urea concentrations the contributions of oligomers decrease with size. This finding is similar to the results obtained in Ref. [1]. Comparing the two urea concentrations, one can see that oligomers larger than dimers are almost negligible at 5 M. Moreover, the dimer concentration is almost twice for 10 M compared to 5 M solution.

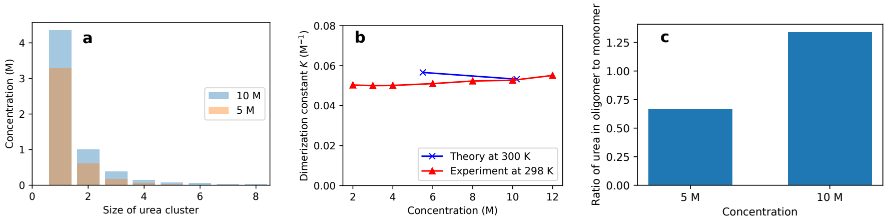

Fig. S1: **Number of urea dimer for various concentrations.** (a) Concentration of urea monomers/ hydrogen-bonded oligomers as a function of size for 10 M and 5 M aqueous urea solutions. The results are obtained from MD simulations. (b) Dimerization constant K at different concentrations from the calculations (at 300 K) and from Ref. [2] (at 298 K). (c) Ratio of urea oligomers to monomers for 5 M and 10 M aqueous urea solutions.

To confirm the validity of our MD simulations, we calculated the equilibrium dimerization constant $K = c_d/c_m^2$ where c_d and c_m are the concentration for dimer and monomer, respectively. The calculated values ($K = 0.056\,\mathrm{M}^{-1}$ and $K = 0.053\,\mathrm{M}^{-1}$ for 5 M and 10 M, respectively) are compared in Fig. S1b with experimental data from Ref. [2] at $T = 298\,\mathrm{K}$. As can be seen, there is good agreement with these values.

The ratio of urea oligomers vs. monomers is shown in Fig. S1c. As can be seen, this ratio is roughly twice as large for 10 M compared to 5 M, indicating a drastic difference between the two urea concentrations. The much larger ratio of urea molecules that are hydrogen-bonded to one another suggests a larger number of rapid proton transfer reactions between urea molecules in 10 M solution and thus

explains the qualitative differences in the respective XAS highlighted in Fig. 2 in the main text between the two concentrations. Note that the contrast between $10\,\mathrm{M}$ and $5\,\mathrm{M}$ is much more pronounced in Fig. S1c than in Fig. S1a (oligomer concentrations) because an oligomer of size n contains n hydrogen-bonded molecules and thus contributes n times to the oligomer vs. monomer ratio.

1.2 Theoretical Time-resolved X-ray Absorption Spectra Results

Figure S2 shows the calculated TRXAS spectra at the carbon and nitrogen K-edges for QM urea dimer with and without proton transfer, and for QM urea monomer with a hydrogen-bonded water molecule in $10\,\mathrm{M}$ aqueous urea solutions. Figures S2a and S2e show the same data as shown in the main text in Fig. 2e and 2g, respectively. Compared to the strong dynamical changes in the TRXAS for trajectories with proton transfer between urea dimers at the carbon absorption resonance (Fig. S2a), almost no dynamical changes are observed for the trajectories where proton transfer does not occur (Fig. S2c). Furthermore, the TRXAS for trajectories involving a single urea and a water molecule does not show any change (Fig. S2e) as no proton transfer occurs here. To validate the selection of the QM region, we also performed calculations with a QM-region involving two neighboring urea molecules and a close-by water molecule. The resulting TRXAS show no significant differences compared to the calculations with just two urea molecules in the QM region.

For the TRXAS at the nitrogen 1s$\rightarrow$valence resonance transition (Figs. S2b, S2d, and S2f), no strong temporal changes are observed. Only for the trajectories with proton transfer (Fig. S2b), a small transient redshift at $\simeq 100\,\mathrm{fs}$ in the resonance position around $398\,\mathrm{eV}$ can be seen.

To analyze the effect of proton transfer, ensemble-averaged integrated absorption cross sections and energy shifts for the carbon 1s$\rightarrow$valence transition are shown in Extended Data Fig. 5 for the QM dimer trajectories with proton transfer. Extended

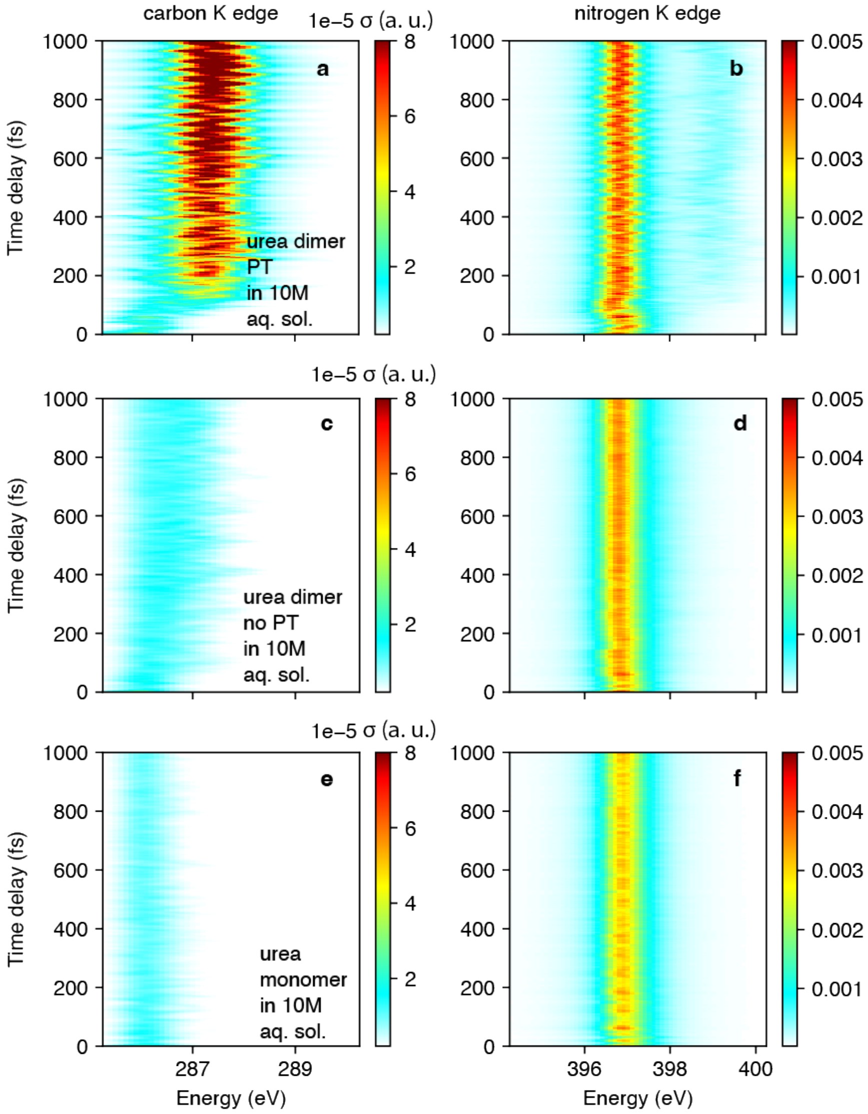

Fig. S2: **Time-resolved X-ray absorption calculations for different urea configurations.** Calculated TRXAS (given by cross sections in a.u.) for 10 M aqueous urea from QM/MM trajectories following HOMO ionization for the 1s→valence resonance absorption transition from the carbon (a, c, e) and nitrogen (b, d, f) core levels. The panels show the calculated TRXAS from trajectories with proton transfer (a and b) and without proton transfer (c and d). Panel (e) and (f) show the TRXAS from calculations that involve a urea molecule and an adjacent water molecule in the QM region.

Data Fig. 5a and Extended Data Fig. 5b are similar to Figs. 4e and 4f in the main text, except that here ensemble-averaged quantities are shown. The sigmoidal fits in Extended Data Fig. 5a and Extended Data Fig. 5b give half-rise times of (134 ± 10) fs and (107 ± 7) fs for the cross section and energy shift, respectively. Consistent with what is discussed for the example trajectory in Fig. 4 in the main text, these ensemble values indicate that the energetic position of the resonance shifts somewhat earlier than the increase in the absorption cross section. In a previous theoretical work, very similar dynamical changes of the TRXAS for urea dimers in vacuum (cyclic conformer) have been observed [3], shown here in Extended Data Fig. 5c and Extended Data Fig. 5d for comparison. As can be seen, the changes in the TRXAS in vacuum occur considerably faster. In particular, we obtain half-rise times of (110 ± 3) fs (from sigmoidal fit) and about 60 fs for the cross section and the energy shift, respectively. This comparison indicates that in the aqueous solution at 300 K, the interaction with the environment and the large number of structural conformations considerably slow down the dynamics leading to the slower temporal changes in the TRXAS.

1.3 Deeper-valence Hole States

Extended Data Fig. 6 shows the binding-energy distribution for molecular orbitals from HOMO to HOMO-6 for neutral urea dimers in the 10 M aqueous solution. This result is similar to the binding-energy distribution for a cyclic dimer in vacuum [3] with HOMO to HOMO-5 lying close to each other in energy within about 3 eV. Due to this proximity in binding energies, also deeper-valence orbitals down to HOMO-5 are likely to be ionized in the experiment. Thanks to the computational efficiency of the Koopmans' theorem approach, our computational model can also address valence holes in orbitals that are more strongly bound than HOMO. These energetically higher valence-ionized states decay rapidly within the first tens of femtoseconds

to the ground state of the ionized system, i.e., into the state with a valence hole in HOMO. As representative examples, we calculated the hole population dynamics for an initial valence hole in HOMO-3 and HOMO-5. The corresponding evolution of populations in the different ionized states (valence hole in HOMO to HOMO-5) are shown in Extended Data Fig. 7. As can be seen from Extended Data Fig. 7a for initial HOMO-3 ionization, the population of the HOMO-3 hole state decays to almost zero in less than $50\,\mathrm{fs}$. The population of the HOMO hole state reaches almost one at about $100\,\mathrm{fs}$. Ionizing even deeper to HOMO-5 initially, it takes longer for the populations to reach the hole in HOMO state. It takes about $150\,\mathrm{fs}$ for the population of the HOMO hole state to reach almost one in this case as seen from Extended Data Fig. 7b. These internal conversion dynamics are reflected in the TRXAS by an absorption resonance initially at a lower energy which shifts over time to higher energies as the valence hole moves from the deeper valence orbital to HOMO (see Fig. 3e in main text).

1.4 Proton Transfer Analysis

We analyzed the temporal progress of proton transfer by calculating the fraction of trajectories in which proton transfer has occurred as a function of time. For this analysis, we consider a proton transfer to have occurred when the distance between the donor-urea hydrogen and the acceptor-urea oxygen is less than 1.25 Å. The percentage of proton transfer as a function of time is shown in Extended Data Fig. 8 for different cases. Extended Data Fig. 8a and Extended Data Fig. 8b show the proton transfer following HOMO and HOMO-3 ionization, respectively. Following HOMO ionization, about 7% of trajectories undergo proton transfer. Most of the proton transfer dynamics are completed before $400\,\mathrm{fs}$. For HOMO-3 ionization, about 17% undergo proton transfer. The proton transfer dynamics continue up to $1\,\mathrm{ps}$, with most

of the proton transfer occurring around $200\,\text{fs}$. The observation that HOMO-3 ionization results in more proton transfer can be explained by the fact that after internal conversion, the ionized molecule has a larger vibrational energy that facilitates to overcome the proton transfer barrier.

Extended Data Fig. 8c shows an artificial scenario where the MM part has been removed from the ionized-dynamics simulation. This setup allows us to highlight the effect of the MM environment. As can be seen from the figure, the ratio of proton transfer trajectories keeps increasing up to $1\,\text{ps}$. About 25% of the trajectories undergo PT within $1\,\text{ps}$. This is clearly different from the results shown in Extended Data Fig. 8a, which includes the MM environment. We explain this observation by the fact that suddenly removing the MM environment leads to a large instantaneous vibrational energy, because the QM cluster is suddenly put into a far-from-equilibrium situation. Moreover, without the liquid environment, any vibrational excess energy in the urea molecule after ionization cannot be dissipated to the surroundings. This excess energy remains in the ionized QM system and helps to overcome the proton transfer barrier.

For further comparison, in Extended Data Fig. 8d we show gas-phase simulation results from Ref. [3]. These calculations have been performed for a cyclic dimer conformer that results in a much larger amount of proton transfer (about 70%). The proton transfer is also considerably faster due to the ideal initial geometrical arrangements of the two urea molecules, which is also reflected in its spectra as discussed earlier for Extended Data Fig. 8c and Extended Data Fig. 3d.

Figure S3 shows the evolution of the proton transfer coordinate, defined as $d_{NH} - d_{OH}$ (the distance between donor-nitrogen and H minus the distance between acceptor-oxygen and H), over time for the QM/MM simulations following HOMO

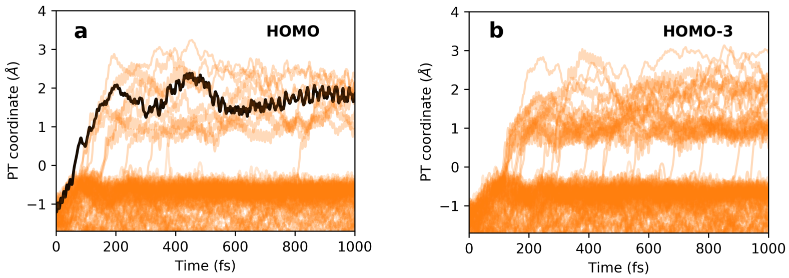

Fig. S3: **Time evolution of the proton transfer coordinate.** proton transfer coordinate in urea dimer, as a function of time, for all QM/MM trajectories in orange following ionization of (a) HOMO and (b) HOMO-3 in a $10\,\mathrm{M}$ aqueous urea solution. The black line in (a) highlights the example trajectory that undergoes proton transfer shown in Fig. 4 of the main text.

and HOMO-3 ionization. A positive value of the coordinate signifies that the proton has been transferred. The figure nicely illustrates how the proton is transferred at different points in time for different trajectories.

1.5 Geometrical-parameter Analysis for Proton Transfer

As described in the main text, we observe that whether or not proton transfer occurs strongly depends on the initial geometrical structure of the dimer that is selected for the QM region in our simulations. Figure S4 shows the fraction of trajectories where the dimer is initially hydrogen bonded via the hydrogen atoms next to the oxygen (H_{proximal}) or via the hydrogen atoms opposite to the oxygen (H_{distal}). Figures S4b and S4c show illustrative examples of the two different dimer structures. As can be seen from the blue bars, both structures occur in similar amounts in aqueous solution. The orange bars indicate the fraction of those trajectories that perform a proton transfer. As can be seen, proton transfer is highly selective for dimer structures that are linked via the H_{proximal} hydrogen atom.

We investigated some geometrical parameters to further highlight the dependence of proton transfer on the dimer structure. Figure S5 shows scatter plots of two geometrical parameters, namely, the oxygen-oxygen distance (d_{O-O}) and the angle

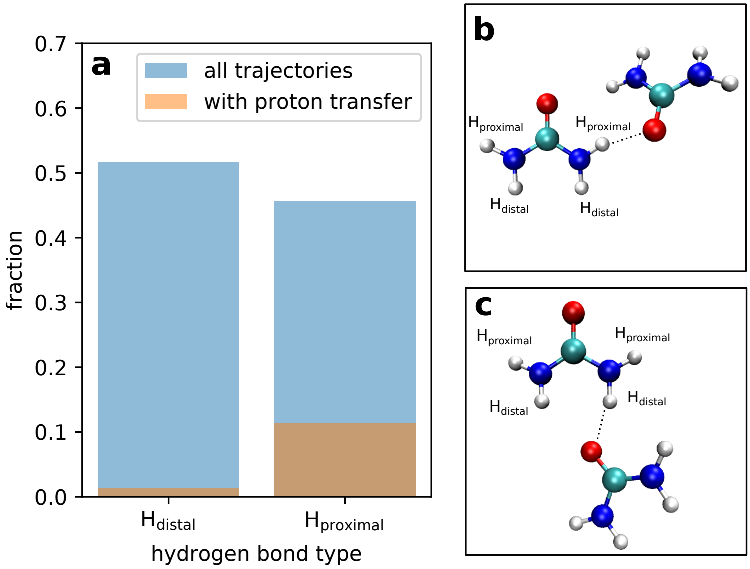

Fig. S4: **Geometry dependence of urea dimer for proton transfer.** (a) Fraction of initial QM/MM dimer structures connected via hydrogen bond with an $H_{proximal}$ or an H_{distal} atom (simulations involving HOMO and HOMO-3 ionization combined). The orange bars represent the fraction of trajectories that undergo proton transfer. Exemplary structures for dimers hydrogen bonded through (b) $H_{proximal}$ or (c) H_{distal} are shown on the right-hand side.

between the carbon-oxygen bond vectors (α_{C-O}) of two ureas in the $10\,\mathrm{M}$ aqueous urea solution. These geometrical parameters for dimers in the equilibrium solution are shown via blue dots in panels (c), (d), and (e). For ionized QM/MM trajectories that undergo proton transfer, the geometrical parameters at the time of proton transfer following initial ionization of HOMO (panel c) and HOMO-3 (panel d), and HOMO ionization without MM (panel e) are shown on top of this distribution as orange dots. HOMO ionization without MM denotes the artificial setup described earlier: the MM environment is stripped off right before ionization. As can be seen from the distribution of the blue dots (panels (c), (d) and (e)), there is a larger variety of initial dimer geometries. The distribution can be roughly clustered into two regions for d_{O-O}<4.3 Å and d_{O-O}>4.3 Å as a consequence of the prevalent relative positions

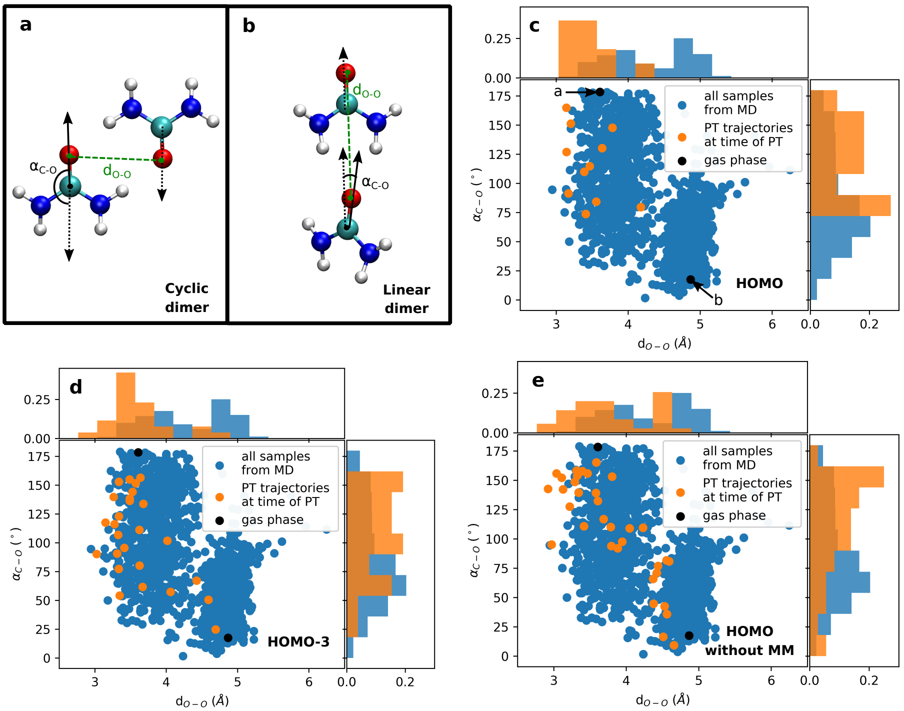

Fig. S5: **Geometrical distribution of urea dimers.** Panels (a) and (b) show two dimer structures in vacuum. Two geometrical parameters are highlighted: the distance between oxygens of the two ureas (d_{O-O}) and the angle between carbon-oxygen bond vectors of the two ureas (α_{C-O}). Panels (c), (d), and (e) show scatter plots in the d_{O-O}-α_{C-O} plane for dimers in the 10 M aqueous solution. The blue dots show the distribution for dimers in equilibrium. The black dots show the parameters for the geometrical conformations of the vacuum structures shown in panels A and B. The orange dots indicate the geometrical conformation at the time when proton transfer occurs following ionization in (c) HOMO and (d) HOMO-3, and (e) following ionization in HOMO, where the MM environment has been removed. Panels (c), (d), and (e) also show normalized histograms for the two employed geometrical parameters on the top and on the left side of each scatter plot.

and orientations of the two urea molecules [4]. For comparison, we also show the two vacuum geometries, namely, the cyclic and the linear dimer geometry, as black dots (the geometries are shown in Figs. S5a and S5b). These vacuum geometries have been obtained by geometry optimization using HF with 6-31+G(d) basis set. The cyclic dimer corresponds to a large α_{C-O} and small d_{O-O}, whereas the linear dimer corresponds to a small α_{C-O} but large d_{O-O}. After HOMO ionization (panel c), proton

transfer preferentially occurs at geometries with relatively small d_{O-O} and at α_{C-O} larger than 75°. This finding is in accordance with the preference of hydrogen bonds via the H_{proximal} hydrogen atoms shown in Fig. S4 as they also fulfill these criteria.

As opposed to the trajectories after HOMO ionization where the described geometrical selectivity is pronounced (Fig. S5c), for the trajectories following HOMO-3 ionization, a few proton transfer reactions can also be seen at geometries with d_{O-O}>4.3 Å and α_{C-O}<75° (see Fig. S5d). For the trajectories where the MM environment has been removed, proton transfer seems to be even less selective with respect to the chosen geometry parameters (Fig. S5e). We attribute these results to the fact that the larger vibrational energy produced via HOMO-3 ionization helps to overcome the proton transfer barrier. The even larger vibrational energy in the scenario where the MM environment has been suddenly stripped off leads to proton transfer at even more diverse geometrical conformations.

The fact that the proton transfer is sensitive to the geometrical configuration of the two participating urea molecules in the solution suggests that the temporal evolution of the measured TRXAS coincides with the hydrogen-bond and self-diffusion dynamics in the solution. In fact, a corresponding hydrogen bond is a necessity for the following proton transfer. In Fig. S6a we evaluated from the pure force-field MD simulations the minimal time t it takes for a urea molecule to form a hydrogen bond via one of its two H_{proximal} hydrogen atoms, which eventually may lead to proton transfer. The blue curve (involving all possible pairs of urea molecules) starts at a vertical offset ($\simeq 20\%$) reflecting the fraction of urea molecules that, on average, have such a hydrogen bond. For the remaining urea molecules, the cumulative fraction of molecules that form such a hydrogen bond exhibits a curve that follows a double-exponential saturation function ($1 - w_0 - w_1 e^{-t/\tau_1} - w_2 e^{-t/\tau_2}$) with fit parameters $w_0 = 0.23 \pm 0.01$, $w_1 = 0.35 \pm 0.01$, $w_2 = 0.42 \pm 0.01$, $\tau_1 = 227 \pm 11\,\text{fs}$, $\tau_2 = 4.1 \pm 0.1\,\text{ps}$. Accordingly, a subset of the urea molecules (35%) forms such a

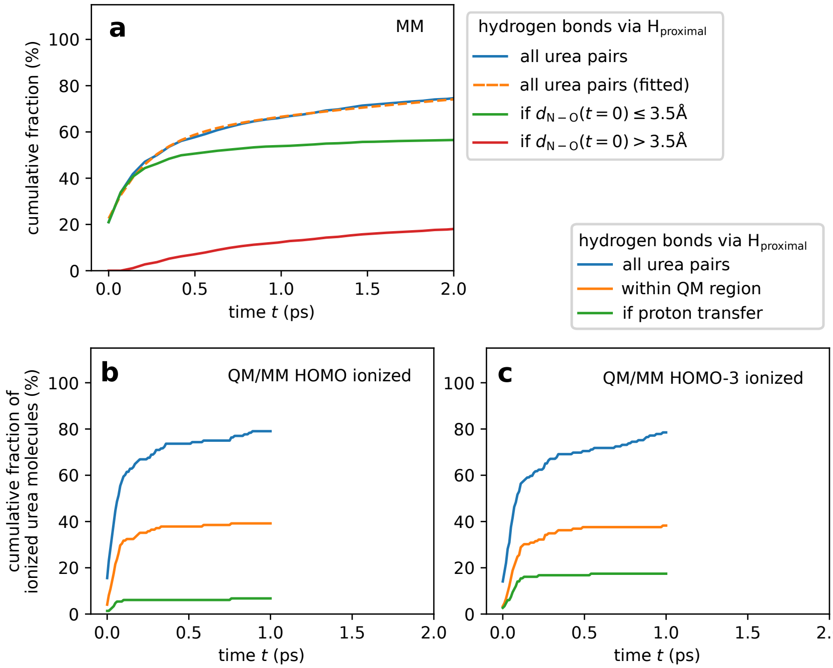

Fig. S6: **Numbers of urea molecules with hydrogen bond after ionization.** Cumulative fractions of urea molecules in a 10M aqueous urea solution that take part in a hydrogen bond via their $\mathrm{H}_{\mathrm{proximal}}$ hydrogen atoms within a given time. (a) Analysis of an MM simulation of a neutral urea solution. Details and results of the fit are given in the text. (b) and (c) Analysis of QM/MM simulations that were initiated with ionization from the HOMO or HOMO-3, respectively.

hydrogen bond relatively fast (within $\simeq 227\,\mathrm{fs}$), whereas another part (42%) requires a considerably longer time ($\simeq 4.1\,\mathrm{ps}$). The green curve in Fig. S6a shows the same analysis but restricted to molecule pairs that are at the reference time $t = 0$ very close to a hydrogen-bond arrangement (i.e., the respective nitrogen-oxygen distance is $\leq 3.5\,\text{Å}$). The red curve shows the cumulative fraction of urea molecules that form a hydrogen bond via its $\mathrm{H}_{\mathrm{proximal}}$ atom, when the intermolecular N-O distance is initially $> 3.5\,\text{Å}$). As can be seen, these two classes clearly separate the fast and the slow time-scale dynamics. Analogous to a similar interpretation of hydrogen-bond dynamics in pure water [5], these two time-scales can be directly linked to

local hydrogen-bond fluctuations among molecules in close vicinity to each other and spatial diffusion dynamics, respectively.

Bearing in mind that this data stems from MD simulations of a neutral (non-ionized) solution, it cannot be expected that it quantitatively matches the situation for ionized urea. We therefore also conducted a similar analysis for the QM/MM MD simulations that consider initial ionization in HOMO (Fig. S6b) or HOMO-3 (Fig. S6c). Here, the analysis is restricted to the urea molecule on which the charge eventually localizes during the QM/MM simulation. Although the statistics are not as good as for the MM data, both figures indicate that the ionized molecule tends to form such hydrogen bonds somewhat faster than in the neutral case. If we further restrict the analysis to those $H_{proximal}$-hydrogen bonds that are formed within the QM region (orange curve), we can conclude that roughly 50% of the ionized urea molecules first form such a bond to a urea molecule in the MM region. In these cases, proton transfer is prohibited in the simulation, which is one of the reasons our simulated TRXAS signal is much weaker than in the experimental data. From those $H_{proximal}$-hydrogen bonds within the QM region, only a fraction leads to proton transfer, as the green curve indicates, where the analysis is restricted to trajectories with proton transfer. Remarkably, after HOMO-ionization, the relative fraction is much lower compared to HOMO-3 ionization indicating that the proton transfer-barrier is easier overcome through the additional vibrational energy provided by HOMO-3 ionization. We note that the respective barriers may be somewhat overestimated in our simulation, resulting in an additional suppression of the number of proton transfer reactions.

Considering that the TRXAS is somewhat delayed due to the involved structural dynamics after the proton transfer, it is worthwhile to point out that the hydrogen-bond dynamics shown in Fig. S6a qualitatively matches the temporal behavior of the changes in the experimental TRXAS in Fig. 3b in the main text: Initially, there is a

rather quick change on the time scale of a few 100 fs stemming from ionized urea molecules that are close to a geometrical conformation allowing for proton transfer to a neighboring urea molecule. This early change in the TRXAS is followed by a slower rise on the times cale of picoseconds due to molecules that are further away from such a configuration. Such an initially rapid reaction rate that slows down to a ps time scale is thus in accordance with what one would expect from a reaction between urea molecules in the 10M solution.

It can be concluded that for short delay times the experimental pump-probe signal can be linked to proton transfer reactions including local dynamical fluctuations of hydrogen bonds. (Notably, the proton transfer dynamics (Extended Data Fig.8a+b) are somewhat slower than the local hydrogen-bond fluctuations (Fig. S6b+c).) Accordingly, our experiment resolves the progress of local chemical reaction dynamics between neighboring molecules in the solution. The probed reaction dynamics can therefore be considered universal for neighboring urea molecules in aqueous urea solutions of any concentration. Only at larger time scales (i.e., several ps), the experimental signal becomes dominated by the spatial diffusion dynamics in the liquid that varies with the urea concentrations.

1.6 Overview of TRXAS Results at the Carbon and Nitrogen Edges

Figure S7 shows the overview of the experimental TRXAS recorded at both the carbon and the nitrogen K-edges. Parts of these data are presented in Figs. 1c, 2a-d and 3a-b of the main text. Here, the complete data is shown, whereby the bleaching of the ground-state signal is visible in dark-blue color and the appearance of additional absorbance from the pumped sample appears in colors ranging from cyan to red. The ground-state bleaching is best visible as a broad feature at 300-305 eV and a

very narrow feature at ∼290 eV, corresponding to the C1s→σ^* and C1s→π^* transitions of aqueous ground-state neutral urea, respectively. The appearance of additional absorbance from the pumped sample at the carbon edge is discussed in the main text. Related time-dependent absorption features are also observed at the nitrogen edge, although the absorption bands are spectrally broader, in general. This holds true both for the pre- and the post-edge features. The ground-state bleaching extends over the region from 403 eV up to the edge of the figure panel (420 eV). The pre-edge region also lacks sharp spectral features, such that it was not discussed in more detail in the main text.

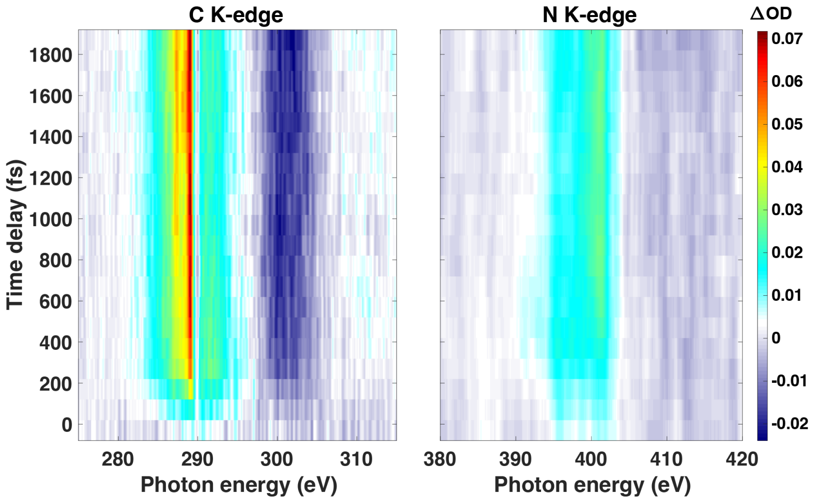

Fig. S7: **Overview of the time-resolved data.** Time-resolved XAS at carbon and nitrogen edges of 10M urea aqueous solution.

Figure S8 shows the depletion-corrected experimental absorbance data at the carbon K-edge. In brief, the experimental difference (ΔOD) spectra are corrected for the ground-state bleaching by adding the ground-state absorption spectrum with a time-dependent coefficient that describes the depletion fraction of the unpumped molecules. Mathematically, this is described by a Heaviside step function, convoluted with the Gaussian cross-correlation function. These depletion-corrected spectra

represent the time-dependent absorbance of the photoexcited sample, devoid of the ground-state bleaching contributions. These spectra highlight the gradual appearance of the absorption band at 289 eV, assigned to the C1s$\rightarrow \pi^*$ transition of the ionized urea molecule, as well as the time-delayed, gradual appearance of the band at 286.5-287.5 eV, assigned to the proton transfer dynamics in the urea dimer. What the depletion-corrected spectra additionally reveal is the decay of the broad absorption band centered around 300 eV. By analogy with the absorption spectrum of neutral ground-state urea, this band is assigned to the C1s$\rightarrow \sigma^*$, transition, i.e., a shape resonance, which is strong in neutral ground-state urea, but appears to vanish following ionization on a $\sim$300-fs time scale.

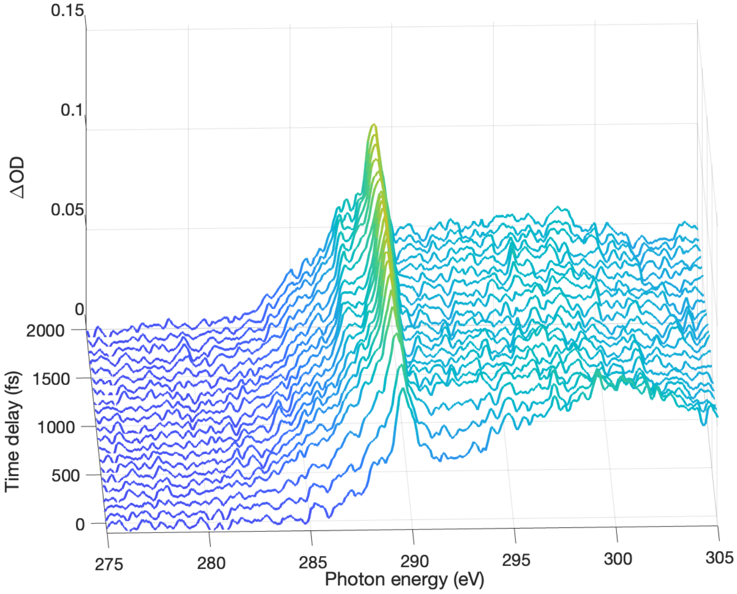

Fig. S8: **Overview of deepletion corrected results.** Depletion-corrected time-resolved XAS at carbon edge of 10M urea aqueous solution.

1.7 Role of the Urea Concentration

We have performed a detailed study of the influence of the urea concentration on the observed dynamics. Extended Data Fig. 9 shows the comparison between the time-resolved carbon-K-edge XAS of 10M, 8M, 5M and 2.5M urea aqueous solutions. In the 10M and 8M urea solutions, there is a strong signature of proton transfer in the urea dimer. At the lower 5M and 2.5M concentrations, there are little-to-no signatures of proton transfer. The obtained results support our assignment of the observed dynamics to the ionization of urea dimers.

1.8 Role of the Pump Wavelength and Peak Intensity

In this section, we report a detailed study of the possible influence of the wavelength and peak intensity of the pump pulses. Importantly, we find that the observed dynamics neither depends on the wavelength (400 nm vs. 266 nm) nor on the employed peak intensities (ranging from 20 TW/cm^2 to 100 TW/cm^2). These observations support our assignment of the observed dynamics to the ionization of urea and exclude a measurable influence of plasma-induced chemistry.

We first discuss the measurements with 400-nm pump pulses, whereby we have systematically varied the pump intensity from its original value of $\sim$100 TW/cm^2 (used for the data reported in the main text) to 80 and 50 TW/cm^2 (Extended Data Fig. 10). The time constants for the appearance of the proton transfer band centered at 287.6 eV were determined to be 360$\pm$60 fs, 330$\pm$90 fs and 370$\pm$60 fs, i.e. identical within the respective error ranges. The independence of the rise times on the pump intensity further confirms that our experiment probes ionization-induced dynamics, as opposed to reactive processes induced by plasma. Figure S9 shows the extracted proton transfer band (after background subtraction) at $\sim$80 TW/cm^2, with a 640$\pm$230 fs rise time which is consistent with the 590$\pm$130 fs rise time at $\sim$100 TW/cm^2

(Fig. 3b of the main text). Due to low SNR, extracting the proton transfer band is not done for lower intensities.

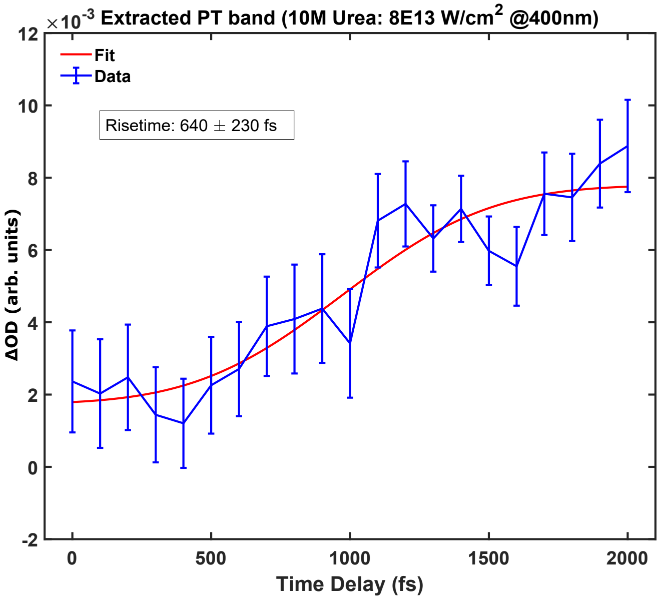

Fig. S9: **Proton transfer rise time for a different pump intensity.** Extracted proton transfer band of 10M aqueous urea solutions performed with ∼30 fs pump pulses centered at 400 nm at ∼80 TW/cm^2 peak intensity.

Measurements with pump pulses centered at 266 nm, instead of 400 nm, have also been performed. Two-photon absorption at 266 nm is energetically equivalent to three-photon absorption at 400 nm, which corresponds to the minimal energy required for ionization of aqueous urea (Ip≈6.9 eV). In these additional measurements, shown in Fig. S10, we have been able to use a 5-times lower peak intensity, compared to the 400-nm measurements presented in the main text. These new conditions drastically reduce the total ionization fraction and plasma density. Nevertheless, we observe essentially identical dynamics, i.e. the same spectral features appearing with the same characteristic rise times. This result demonstrates that the observed dynamics are not caused by the plasma formed by multiphoton ionisation of water

because, if this were the case, the observed time constants would change based on the very different plasma densities reached under 266 nm vs. 400 nm pumping.

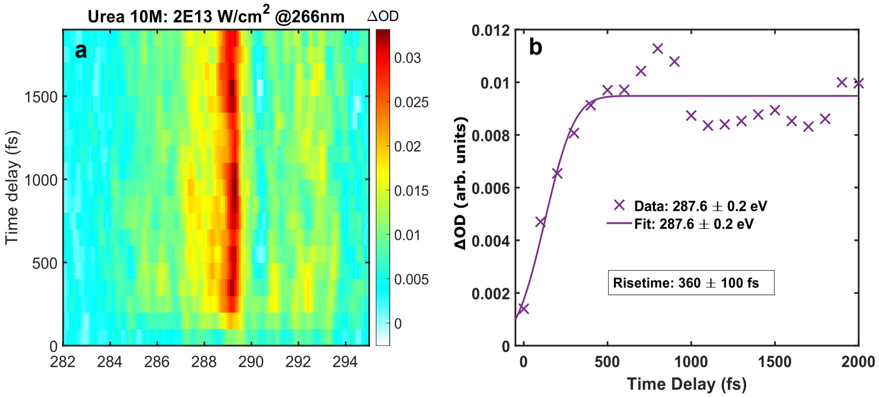

Fig. S10: **Proton transfer dynamics for 266 nm pump.** Time-resolved XAS at the carbon K-edge of 10M aqueous urea solutions performed with 40 fs pump pulses centered at 266 nm with a peak intensity of 20 TW/cm^2.

The laser intensities/wavelengths used in the experiments shown in Extended Data Fig. 9, Fig. S9 and S10 range from ~20 TW/cm^2 at 266 nm till ~100 TW/cm^2 at 400 nm. In all cases, the same spectral features assigned to the proton transfer in ionized urea dimers are observed with the same time constants. This does not imply that ionization of water does not occur. However, as reported by Z. Loh *et al.* [6] and N. Linz *et al.* [7], a minimum pump intensity of around ~100 TW/cm^2 is required to multiphoton ionize liquid water. Finally, the measurements presented in this section also establish that the carbon pre-edge resonance assigned to proton transfer in ionized urea dimers is observable well below the threshold intensity for the ionization of liquid water, excluding dynamics induced by the ionization of water as a significant contributor to the observed proton transfer.

1.9 Results on Aqueous Acetonitrile

The proton transfer initiated via multiphoton ionization in urea solution takes place with significant probability only at high concentrations. As a counter-example we illustrate the distinct lack of such a process in aqueous acetonitrile solution and the corresponding spectral features in time-resolved XAS of aqueous acetonitrile solution as shown in Fig. S11 at carbon and nitrogen K edges. The transient signal rises in the first 25 fs due to acetonitrile cation formation and then stays constant throughout 100 fs delays with absence of the characteristic proton-transfer signatures.

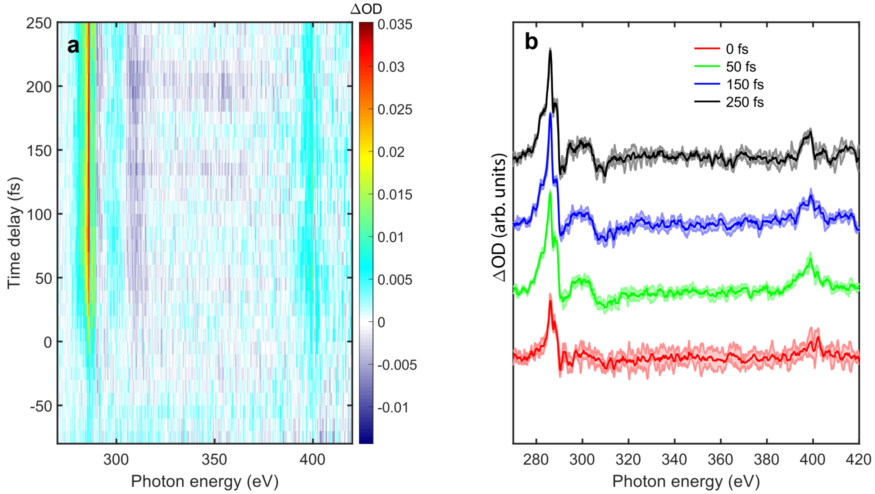

Fig. S11: **Time-resolved XAS results of aqueous acetonitrile for comparison.** TR-XAS results of ∼9.6M aqueous acetonitrile solution following 400 nm pumping recorded under similar experimental conditions as for the urea solutions (Figs. 2 and 3 of the main text). Left: Differential absorbance of the acetonitrile solution from -80 fs till 250 fs. Right: spectra at various time delays showing insignificant spectral changes beyond delays of 50 fs.

References

[1] Idrissi, A., Damay, P., Yukichi, K. & Jedlovszky, P. Self-association of urea in aqueous solutions: A Voronoi polyhedron analysis study. *J. Chem. Phys.* **129** (16), 164512 (2008) .

[2] Stokes, R. H. Thermodynamics of Aqueous Urea Solutions. *Australian Journal of Chemistry* **20** (10), 2087–2100 (1967) .

[3] Shakya, Y., Inhester, L., Arnold, C., Welsch, R. & Santra, R. Ultrafast time-resolved x-ray absorption spectroscopy of ionized urea and its dimer through ab initio nonadiabatic dynamics. *Structural Dynamics* **8** (3), 034102 (2021) .

[4] Stumpe, M. C. & Grubmüller, H. Interaction of urea with amino acids: Implications for urea-induced protein denaturation. *Journal of the American Chemical Society* **129** (51), 16126–16131 (2007) .

[5] Luzar, A. & Chandler, D. Hydrogen-bond kinetics in liquid water. *Nature* **379** (6560), 55–57 (1996) .

[6] Loh, Z.-H. *et al.* Observation of the fastest chemical processes in the radiolysis of water. *Science* **367** (6474), 179–182 (2020) .

[7] Linz, N., Freidank, S., Liang, X.-X. & Vogel, A. Wavelength dependence of femtosecond laser-induced breakdown in water and implications for laser surgery. *Physical Review B* **94** (2), 024113 (2016) .
